# Successful Treatment of Acquired von Willebrand Syndrome in a Rare Case of Nodal Marginal Zone Lymphoma With Rituximab Therapy: A Case Report

**DOI:** 10.7759/cureus.91158

**Published:** 2025-08-28

**Authors:** Gai Matsumura, Sayaka Ohno, Shun Hasegawa, Ryo Shimizu

**Affiliations:** 1 Department of Hematology, Asahi General Hospital, Chiba, JPN; 2 Department of Clinical Pathology, Asahi General Hospital, Chiba, JPN

**Keywords:** acquired von willebrand syndrome, bleeding disorders, m-protein, nodal marginal zone lymphoma, rituximab

## Abstract

Nodal marginal zone lymphoma (NMZL) is an uncommon, mature B-cell lymphoma, rarely associated with acquired von Willebrand syndrome (AVWS), a bleeding disorder caused by von Willebrand factor (VWF) defects secondary to lymphoproliferative disorders (LPDs).

We report a 68-year-old woman with longstanding Sjögren's syndrome, presenting with persistent oral bleeding, anemia, prolonged activated partial thromboplastin time (APTT, 50.5 seconds), and IgA-λ monoclonal gammopathy. Further investigation revealed severely decreased VWF ristocetin cofactor activity (VWF:RCo, 16%). Imaging revealed systemic lymphadenopathy, and a lymph node biopsy confirmed NMZL. The patient was treated with four weekly doses of rituximab, resulting in cessation of bleeding, improvement of coagulation parameters, resolution of anemia, and significant lymphadenopathy regression - consistent with complete response by computed tomography (CT) per Lugano 2014. This case highlights the diagnostic challenges of AVWS associated with NMZL and demonstrates rituximab's efficacy in treating both lymphoma and AVWS.

## Introduction

Nodal marginal zone lymphoma (NMZL) is a distinct and uncommon subtype of mature B-cell lymphoma, constituting less than 10% of marginal zone lymphomas (MZLs) [1,2]. The diagnosis is restricted to cases where the disease is confined to lymph nodes, without evidence of extranodal or splenic lesions. Patients often present with non-bulky, advanced-stage disease, and bone marrow infiltration is observed in about 30% of cases. While NMZL typically follows an indolent course with a good prognosis, the presence of B symptoms or advanced disease can lead to systemic lymphadenopathy, cytopenia, or, on rare occasions, autoimmune manifestations [2].

Acquired von Willebrand syndrome (AVWS) is a rare bleeding disorder caused by defects in von Willebrand factor (VWF), an acquired condition that mimics the features of congenital von Willebrand disease and often develops secondary to underlying conditions, like a lymphoproliferative disorder (LPD) [3,4]. The association between AVWS and MZL, however, is exceptionally uncommon, with very few documented cases arising from NMZL [5,6]. This rarity poses a considerable challenge for both diagnosis and management. Reporting such cases is, therefore, essential for deepening the clinical understanding of this disease combination and for informing effective therapeutic strategies.

While the efficacy of rituximab for AVWS secondary to LPDs can be variable, successful outcomes, including sustained remission, have been documented in individual cases [7,8]. Building on these observations, we report a case of NMZL-associated AVWS that responded favorably to rituximab. Our report contributes valuable data to the discussion of therapeutic options for this rare comorbidity.

This article has been submitted in abstract form to the 86th Annual Meeting of the Japanese Society of Hematology (October 10-12, 2025) and is currently under review.

## Case presentation

A 68-year-old woman with a 35-year history of Sjögren's syndrome, which had been monitored without specific treatment, and no prior personal or family history of bleeding disorders was referred to our Hematology Department. Her blood type is A. The referral was prompted by the incidental findings of a prolonged activated partial thromboplastin time (APTT) and an IgA-λ monoclonal protein. For the three weeks prior to presentation, she had been experiencing intermittent oral bleeding following a dental extraction. On physical examination, active oozing of blood from the gingiva and bilateral inguinal lymphadenopathy were the only significant findings.

Evaluation of the patient's progressive anemia revealed a low hemoglobin level, with otherwise normal white blood cell and platelet counts (Table 1). Coagulation studies confirmed a prolonged APTT, which prompted specialized VWF analysis. This revealed the characteristic findings of AVWS type 2M: a markedly elevated VWF antigen (VWF:Ag) level, severely decreased VWF ristocetin cofactor activity (VWF:RCo), and a regular multimeric pattern. Separately, serum protein analysis identified a high concentration of an IgA-λ monoclonal protein and an elevated polyclonal IgG level (Table 1). A subsequent bone marrow examination for these protein abnormalities revealed a normocellular marrow with 15,800 nucleated cells/μL. Plasma cells comprised 1.0% of all nucleated cells, and no abnormal lymphoid infiltration was detected by morphology or flow cytometry. These findings were consistent with monoclonal gammopathy of undetermined significance (MGUS), without evidence of a primary LPD or multiple myeloma.

**Table 1:** Baseline laboratory data at presentation

Laboratory Data	Patient Values	Reference Range
White blood cells	4.1 × 10^3^/μL	3.3-8.6 × 10^3^/μL
Red blood cells	3.63 × 10^6^/μL	3.86-4.92 × 10^6^/μL
Hemoglobin	8.4 g/dL	11.6-14.8 g/dL
Hematocrit	28.50%	35.1-44.4%
Platelets	17.2 × 10^4^/μL	15.8-34.8 × 10^4^/μL
Prothrombin time	15 sec	10-13.5 sec
Activated partial thromboplastin time	50.5 sec	24-34 sec
Factor VIII (FVIII) assay	25%	60-150%
von Willebrand factor antigen (VWF:Ag)	8,620%	50-155%
von Willebrand factor ristocetin cofactor activity (VWF:RCo)	16%	60-170%
The von Willebrand factor multimer assay		
Large	Positive	Positive
Medium	Positive	Positive
Small	Positive	Positive
IgG	3,192 mg/dL	861-1,747 mg/dL
IgA	4,036 mg/dL	93-393 mg/dL
IgM	146 mg/dL	50-269 mg/dL
Serum immunofixation electrophoresis	IgA-λ	-

**Figure 1:**
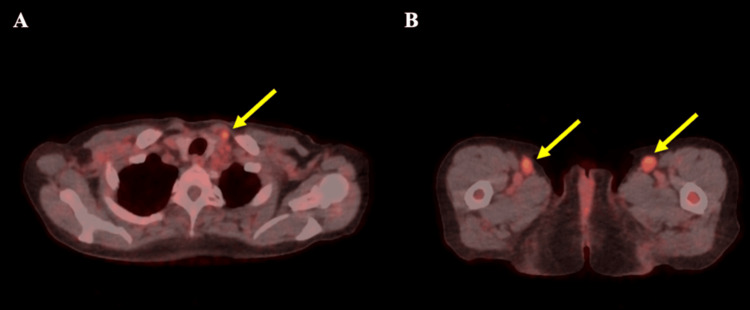
FDG-PET/CT images at diagnosis (A) Axial image showing a hypermetabolic supraclavicular lymph node (yellow arrow). (B) Axial image showing bilateral hypermetabolic inguinal lymph nodes (yellow arrows). PET-CT, positron emission tomography-computed tomography; FDG, fluorodeoxyglucose

Despite the benign bone marrow findings, a positron emission tomography-computed tomography (PET/CT) scan revealed hypermetabolic lymphadenopathy in the mediastinum, supraclavicular, and inguinal regions, prompting an inguinal lymph node biopsy (Figure 1).

**Figure 2:**
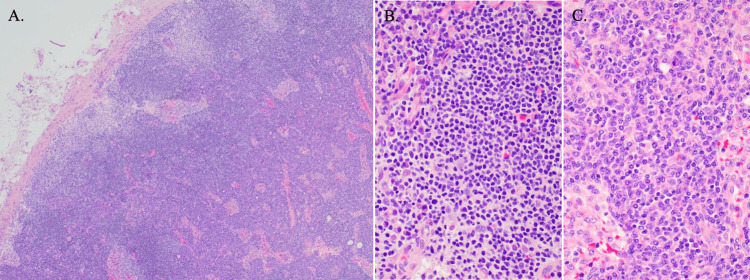
Histopathologic features of the biopsied inguinal lymph node (Hematoxylin and Eosin stain) (A) The follicular structure of the lymph node is obscured due to expansion of the marginal zone and an increase in plasmacytoid cells (original magnification, ×4). (B) In the peripheral region, small lymphocytes with small nuclei and slightly pale cytoplasm predominate (original magnification, ×40). (C) In the central region, plasmacytoid lymphocytes with granular chromatin, round nuclei, and basophilic cytoplasm predominate (original magnification, ×40).

**Figure 3:**
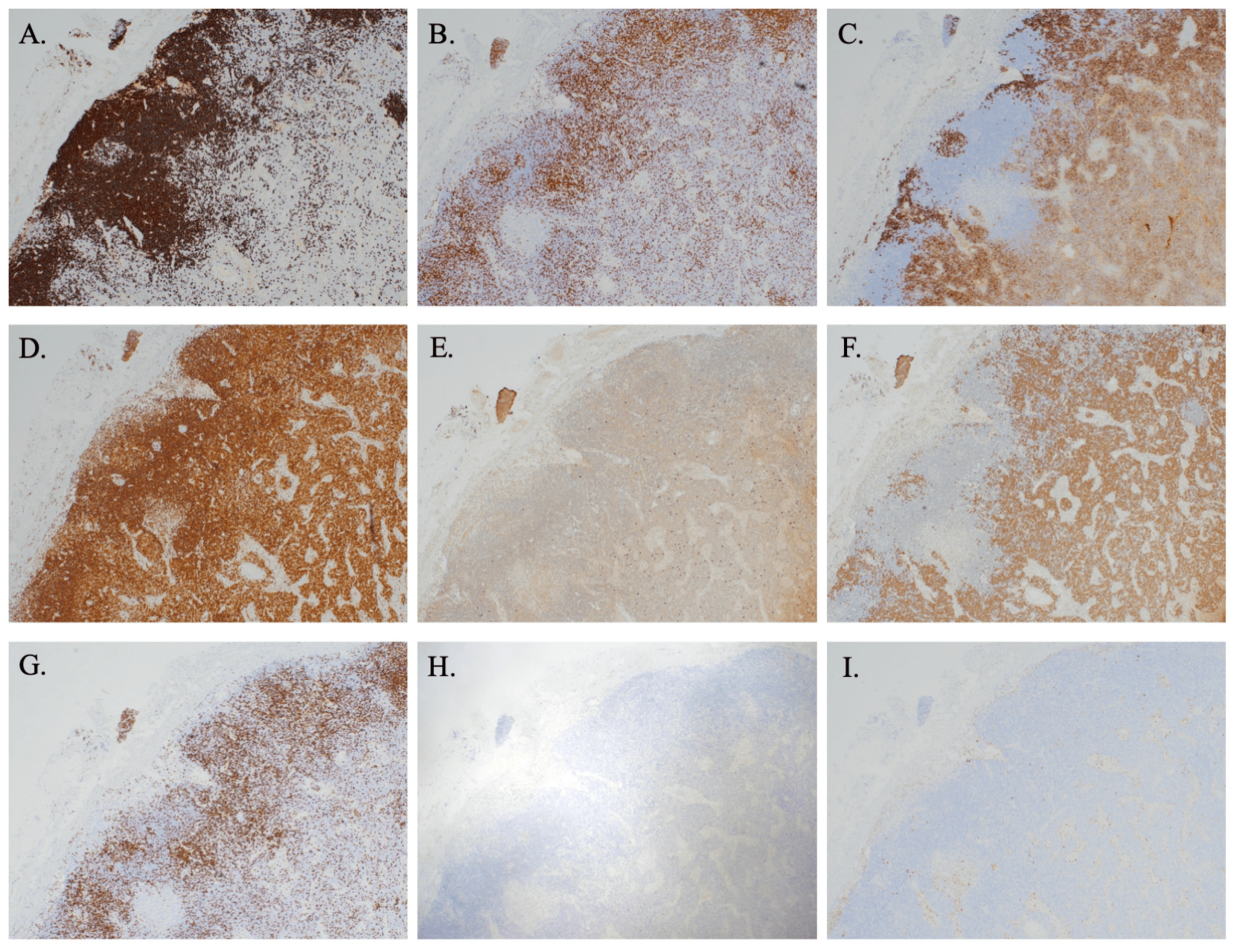
Immunohistochemical staining of the biopsied inguinal lymph node (original magnification ×4) (A) The peripheral small lymphocytes are strongly positive for CD20. (B) Both neoplastic cell populations show positivity for BCL-2. (C) The central plasmacytoid cells are positive for CD138. (D) A background population of reactive T-cells is highlighted by CD3. (E) Staining for κ-light chain is negative. (F) Staining for λ-light chain is positive. (G) The neoplastic cells are negative for CD5. (H) The neoplastic cells are negative for CD10. (I) The neoplastic cells are negative for cyclin D1.

The pathological examination of the inguinal lymph node revealed that the follicular structure was obscured due to the enlargement of the marginal zone and an increase in plasmacytoid cells, as observed through Hematoxylin and Eosin (HE) staining and immunohistochemical staining (Figures 2-3). In the peripheral region of the lymph node, there was a predominant increase in small lymphocytes with pale cytoplasm, characterized as CD20+ and BCL2 (focal+), while the CD138 marker was negative. In the central region, there was an increase in plasmacytoid cells that were CD20- and positive for CD138 and BCL2 (focal+) (Figures 3A-3C). The plasmacytoid cells displayed a clear κ < λ light chain restriction, as demonstrated by in situ hybridization, leading to a diagnosis of low-grade B-cell lymphoma with plasma cell differentiation (Figures 3E-3F). Immunohistochemical staining showed negativity for CD5, CD10, and cyclin D1 (Figures 3G-3I). Additionally, no MYD88 mutations were detected by reverse transcription polymerase chain reaction (RT-PCR). Ultimately, the diagnosis was confirmed as NMZL, which excluded chronic lymphocytic leukemia (CLL), small lymphocytic lymphoma (SLL), follicular lymphoma (FL), and mantle cell lymphoma (MCL).

**Figure 4:**
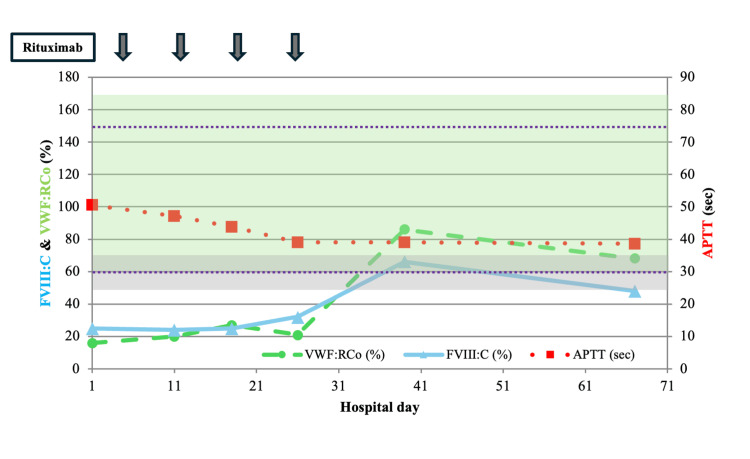
Clinical course and hemostatic parameters following rituximab treatment Weekly rituximab monotherapy (375 mg/m² ×4) led to a robust increase in plasma VWF:RCo (normal range shown in shaded green area; 60-170%), a more modest improvement in FVIII:C levels (reference interval shown with horizontal dashed purple lines; 60-150%), and a trend toward normalization of APTT (normal range shown in shaded gray area; 24-34 sec). Arrows indicate the timing of rituximab administration. APTT: activated partial thromboplastin time, FVIII:C: factor VIII activity, VWF:RCo: von Willebrand factor ristocetin cofactor activity

Due to recurrent oral mucosal bleeding that required outpatient red blood cell transfusions, treatment for NMZL-associated AVWS was initiated. The patient was hospitalized and received four weekly infusions of rituximab (375 mg/m²). Although she required red blood cell transfusions during the treatment course, her mucosal bleeding ceased after the final rituximab administration, and her APTT and VWF:RCo levels improved (Figure 4).

No adverse events related to rituximab were observed, and she was discharged transfusion-independent. On follow-up, VWF:RCo remained within the reference range, and CT imaging demonstrated complete resolution of measurable disease, with all lymph nodes regressing to ≤1.5 cm, no extranodal disease, and no new lesions; per the Lugano 2014 CT-based criteria [9], this constitutes a complete response. PET/CT was not performed. Over follow-up, VWF:Ag measured by latex immunoassay (LIA) varied substantially (1,470-6,730%), while the IgA-λ monoclonal protein persisted, whereas VWF:RCo remained within the reference range. As of six months post-treatment, she is asymptomatic, with no recurrent bleeding, and continues outpatient follow-up.

## Discussion

This case highlights two principal findings: first, the development of AVWS as a rare complication of NMZL, further characterized by an atypical type 2M profile without the loss of high-molecular-weight (HMW) multimers. Second, it demonstrates the successful and complete resolution of the severe bleeding diathesis with rituximab monotherapy, which concurrently induced remission of the underlying lymphoma. These findings are significant, as they contribute valuable information to the limited literature on this specific disease combination and offer insights into effective management strategies.

The association of AVWS with LPDs is a well-established phenomenon [5,10]. Several cases of AVWS associated with various subtypes of MZL have now been reported (Table 2) [6,7,11-15]. Among these cases, AVWS associated with NMZL is rare, with only one definitive case previously documented [15]. This contrasts with other subtypes, such as splenic marginal zone lymphoma (SMZL), where AVWS is more frequently reported and often linked to splenomegaly and specific mechanisms, such as aberrant glycoprotein Ib expression on tumor cells [6,12]. Therefore, the presentation of AVWS in a patient with NMZL - particularly without splenic involvement - represents a significant clinical observation.

**Table 2:** Summary of reported cases of AVWS in MZL subtypes MALT, mucosa-associated lymphoid tissue; AVWS, acquired von Willebrand syndrome; MZL, marginal zone lymphoma

Author (Year) (Ref)	Subtype	AVWS Mechanism	Therapy	AVWS Outcome
Tefferi et al. (1997) [6]	MZL, splenic	Type 2	Splenectomy	Overall response
Iwabuchi et al. (2011) [7]	MZL, thymic MALT	Type 2A	Rituximab	Overall response
Kumar et al. (2003) [11]	MZL, splenic	Type 2	Splenectomy + chemotherapy	Overall response
Komeno et al. (2017) [12]	MZL, splenic	Type 2A	Rituximab	No response
Biondo et al. (2012) [13]	MZL, gastric MALT	Not mentioned	Rituximab	Overall response
Owari et al. (2023) [14]	MZL, mediastinal MALT	Mixture of type 1/2	Rituximab	Overall response
Fidalgo et al. (2017) [15]	MZL, nodal	Type 2B	Multidrug therapy	Overall response
Our Case	MZL, nodal	Type 2M-like	Rituximab	Overall response

This case presented a diagnostic challenge due to its atypical laboratory profile, which is consistent with a type 2M-like AVWS [3]. Unlike typical AVWS, which is often characterized by a loss of HMW VWF multimers, our patient's multimeric analysis was normal. This pattern, while uncommon, has also been reported in other cases of lymphoma-associated AVWS, including a recent report describing normal multimers and suggesting a distinct, underrecognized pathophysiological mechanism in these patients [16], as well as in AVWS associated with monoclonal gammopathies [17]. Although M-proteins, such as IgA in our case, have been proposed to interfere with VWF function (particularly binding to platelet glycoprotein Ib), our patient’s AVWS resolved despite persistently high IgA levels. This suggests that the monoclonal IgA itself was unlikely to be the primary cause, implicating the underlying lymphoma as the key driver, consistent with previous reports [14].

In addition, the M-protein may directly interfere with the VWF:Ag assay. The remarkably high VWF:Ag by LIA could represent a measurement artifact - a phenomenon described by Okamoto et al., who showed that patient-derived IgG produced non-specific reactivity detected by LIA but not by enzyme-linked immunosorbent assay (ELISA) [18]. In our case, the persistently elevated and widely fluctuating LIA-derived VWF:Ag (1,470-6,730%), despite clinical and laboratory remission, together with the persistence of the IgA-λ M-protein, is compatible with ongoing assay interference and warrants cautious interpretation of antigen values.

The management of AVWS secondary to an LPD primarily focuses on treating the underlying malignancy, as this offers the best chance for durable hemostasis [5]. The resolution of AVWS can only be sustained through the successful management of the underlying LPDs, emphasizing the need to focus therapeutic efforts on the primary malignancy. Rituximab, which targets the CD20-positive B-cell clone, is a key therapeutic option for LPD-associated AVWS [5]. Accordingly, rituximab monotherapy was selected for our patient’s CD20-positive NMZL. The treatment proved highly effective, leading to remission of the lymphoma and complete resolution of the AVWS. This successful outcome is consistent with other case reports in which rituximab-based therapy led to resolution of AVWS in patients with various subtypes of MZL, including mucosa-associated lymphoid tissue (MALT) lymphoma [7,14]. This contrasts with cases in which rituximab was ineffective because the underlying lymphoma was refractory [12].

An intriguing aspect of our case was the persistence of high IgA levels despite clinical remission. This supports the hypothesis that the AVWS was directly linked to the activity of the CD20-positive lymphoma cells, rather than to the circulating M-protein produced by CD20-negative plasma cells - a notion reinforced by rituximab’s known limited efficacy in plasma cell-driven disorders [5].

## Conclusions

In conclusion, this case offers two key clinical takeaways. First, NMZL should be recognized as a rare but important cause of AVWS, requiring a high index of suspicion in patients with unexplained bleeding. Second, this report supports rituximab monotherapy as an effective treatment, capable of resolving the coagulopathy by successfully treating the underlying lymphoma. This experience underscores that early diagnosis of this rare comorbidity and consideration of a targeted therapeutic approach are essential for optimizing patient outcomes.
